# Engineering the future of medicine: Natural products, synthetic biology and artificial intelligence for next‐generation therapeutics

**DOI:** 10.1002/ctm2.70146

**Published:** 2025-01-24

**Authors:** Emre F. Bülbül, Helge B. Bode, Steven Schmitt, Kenan A. J. Bozhüyük

**Affiliations:** ^1^ Synthetic Biology of Microbial Natural Products Helmholtz Institute for Pharmaceutical Research Saarland (HIPS), Helmholtz Centre for Infection Research (HZI), PharmaScienceHub (PSH) Saarbrücken Germany; ^2^ Department of Natural Products in Organismic Interactions Max Planck Institute for Terrestrial Microbiology Marburg Germany; ^3^ Department of Biosciences Molecular Biotechnology Goethe‐University Frankfurt Frankfurt Germany; ^4^ Center for Synthetic Microbiology (SYNMIKRO) Phillips University Marburg Marburg Germany; ^5^ Department of Chemistry Phillips University Marburg Marburg Germany; ^6^ LOEWE Centre for Translational Biodiversity Genomics (LOEWE‐TBG) & Senckenberg Gesellschaft für Naturforschung Frankfurt Germany; ^7^ Myria Biosciences AG, Tech Park Basel Basel Switzerland

**Keywords:** AI, bioengineering, natural products, synthetic biology

## Abstract

The e ***X*** change ***U*** nit between ***T*** hiolation domains approach and artificial intelligence (AI)‐driven tools like *Synthetic Intelligence* are transforming nonribosomal peptide synthetase and polyketide synthase engineering, enabling the creation of novel bioactive compounds that address critical challenges like antibiotic resistance and cancer. These innovations expand chemical space and optimize biosynthetic pathways, offering precise and scalable therapeutic solutions. Collaboration across synthetic biology, AI, and clinical research is essential to translating these breakthroughs into next‐generation treatments and revolutionizing drug discovery and patient care.

## 1 INTRODUCTION

Natural products have long served as a foundation for medicine, leading to transformative therapies such as penicillin, cyclosporin, and taxol that address critical challenges in infectious diseases and cancer. Among these, nonribosomal peptides and polyketides, along with their hybrids, stand out for their immense therapeutic potential.^1^ These bioactive compounds are synthesized by modular megasynth(et)ases, such as nonribosomal peptide synthetases (NRPSs),^2^ polyketide synthases (PKSs),^3^ and hybrids thereof—which assemble structurally complex molecules through sequential incorporation of diverse extender units.

NRPSs are able to use more than 500 extender units (proteinogenic and non‐proteinogenic amino acids),^2^ while PKSs utilize derivatives of malonyl‐CoA, such as methylmalonyl‐CoA,^3^ to build molecular frameworks. Both systems function like molecular assembly lines, where modules in the enzyme activate, incorporate, and process extender units. In NRPSs, the adenylation (A) domain selects and activates amino acid extender units, while in PKSs, the acyltransferase (AT) domain performs this role for CoA derivatives. Activated extender units are transferred to carrier proteins—thiolation (T) domains in NRPSs and acyl carrier proteins (ACP) in PKSs—before being linked to the growing chain by condensation (C) domains in NRPSs or ketosynthase (KS) domains in PKSs.

The modular architecture of NRPSs and PKSs establishes a direct correlation between the sequence of domains in their assembly lines and the structure of the biosynthesized molecules.^2^, ^3^ This relationship enables the prediction of molecular structures based on DNA sequences. Furthermore, this modularity allows for the rational substitution of genetic building blocks—DNA fragments encoding specific enzymatic domains—offering the potential to engineer enzymes capable of producing novel compounds.

Despite this theoretical flexibility, the structural complexity of NRPSs and PKSs, along with the tightly coordinated interactions between their domains, makes reprogramming these systems challenging.^4^ Traditional chemical synthesis often cannot replicate the diversity and intricacy of these natural products, underscoring the need for innovative bioengineering approaches to unlock their full potential.

To overcome these limitations and build on our earlier works,^5^, ^6^ we developed the e ***X*** change ***U*** nit between ***T*** hiolation domains (*XUT*) approach.^7^ Inspired by natural evolution, *XUT* enables the targeted replacement of genetic building blocks within NRPS and NRPS‐PKS systems, facilitating modular recombination and significantly expanding chemical space. By designing recombinant enzymes that produce novel molecules, *XUT* lays a robust foundation for innovative drug discovery. Coupled with our *Synthetic Intelligence* platform—an artificial intelligence (AI)‐driven system that optimizes biosynthetic pathways—*XUT* accelerates the development of next‐generation therapies to address critical challenges such as antibiotic resistance and cancer.

### 1.1 Unlocking NRPS engineering: the *XUT* approach

**FIGURE 1:**
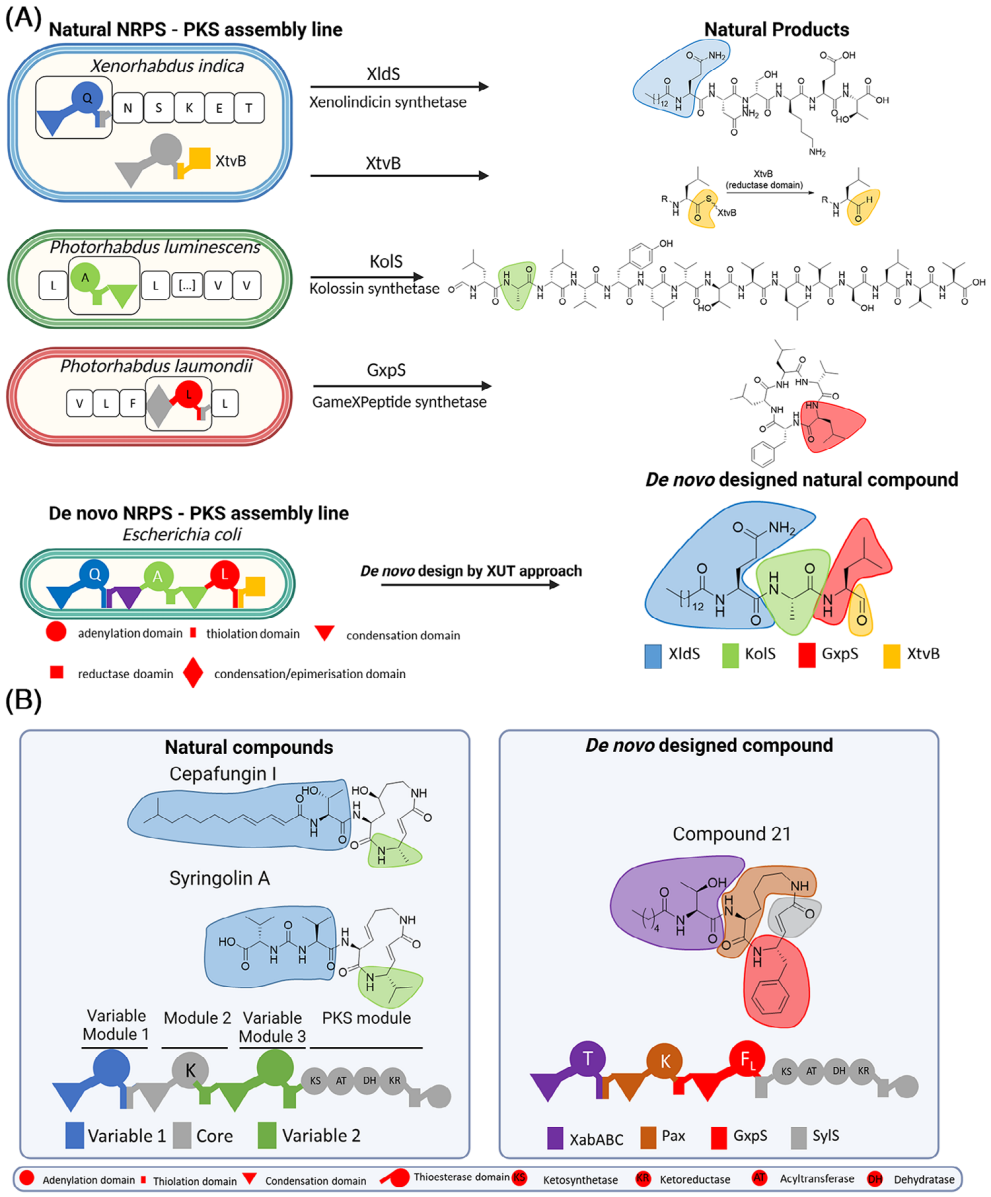
Natural and de novo nonribosomal peptide synthetase‐polyketide synthase (NRPS‐PKS) assembly lines. (A) Natural NRPS‐PKS systems in different organisms produce bioactive compounds. These pathways use adenylation (A), thiolation (T), condensation (C) and additional domains such as the reductase domain to create diverse natural products. (B) Using the e ***X*** change ***U*** nit between ***T*** hiolation domains (*XUT*) approach, NRPS‐PKS modules from different systems were recombined in *Escherichia coli* to produce compound 21, a novel syrbactin derivative.

The *XUT* is a transformative approach in NRPS and NRPS‐PKS engineering, addressing structural complexity and enabling seamless module swapping while preserving catalytic activity. This strategy facilitates the rapid creation of bioactive compounds that are challenging or sometimes even inaccessible through conventional methods (Figure 1).

Using *XUT*, we successfully engineered over 50 novel peptides and peptide‐polyketide hybrids, including a proteasome inhibitor active against the yeast 20S proteasome core (Figure 1A).^7^ This proof‐of‐concept experiment highlights *XUT's* potential to create tailor‐made bioactive molecules for therapeutic applications.

Building on these results, we developed NRPS‐PKS hybrid systems, leveraging the modularity of NRPS and the versatility of PKS to produce syrbactin derivatives (Figure 1B).^8^ Syringolin A and cepafungin I, both naturally occurring compounds, serve as examples of the structural diversity achieved by natural biosynthetic pathways. In contrast, compound 21, a de novo‐designed molecule, incorporates tailored structural modifications that distinguish it from its natural counterparts. These derivatives exhibited potent inhibitory activity against immunoproteasome subunits β1i and β5i.^8^ Targeting the immunoproteasome, a critical regulator of immune responses and cancer, these hybrids show initial promise for treating cancer and autoimmune disorders. Ongoing studies aim to further optimize their structures and evaluate their clinical potential.

### 1.2 Next‐generation therapies: addressing antibiotic resistance and beyond

Antimicrobial resistance is a global threat, diminishing antibiotic effectiveness. Novel antibiotics like odilorhabdin, which disrupts bacterial protein synthesis through a unique mechanism, are crucial. By reprogramming the odilorhabdin biosynthetic pathway, we established a platform for creating derivatives with enhanced properties, offering a powerful alternative to traditional chemical synthesis.^9^

Beyond antibiotics^9^ and cancer therapy,^8^ megasynth(et)ases hold promise for treating autoimmune, neurodegenerative, and metabolic disorders. Innovations in synthetic biology, such as the *XUT* approach, combined with AI, are paving the way for highly targeted therapies with minimal off‐target effects. These advancements are unlocking next‐generation treatments with the potential to transform patient care.

### 1.3 Call to action

Recent advances in protein engineering and AI‐driven drug discovery are transforming therapeutic development, yet their translation into effective treatments requires interdisciplinary collaboration. The 2024 Nobel Prize in Chemistry recognized breakthroughs in protein science: David Baker's novel protein designs and AlphaFold's predictive capabilities by Demis Hassabis and John Jumper. These tools empower researchers to model interactions between engineered compounds and therapeutic targets, prioritizing molecules with optimized therapeutic potential.

Building on these advancements, *Synthetic Intelligence*,^10^ an AI‐ and data‐driven synthetic biology platform technology developed in collaboration with Myria Biosciences AG, is currently in a promising stage of development. The platform aims to fundamentally revolutionize the drug discovery process by utilizing advanced computational models to design and optimize biosynthetic pathways capable of producing novel, disease‐specific molecules. Although not yet fully mature, *Synthetic Intelligence* seeks to address critical bottlenecks in drug development and enable the scalable and efficient translation of tailored compounds into clinical applications.

Clinicians play a vital role in validating these compounds and assessing their impact in real‐world settings. By uniting AI, structural biology, bioengineering, and clinical expertise, we can accelerate the development of next‐generation therapies, addressing urgent challenges like antibiotic resistance and cancer with precision and efficiency.

## 2 CONCLUSION

The *XUT* approach and AI‐driven tools like *Synthetic Intelligence* are transforming NRPS and PKS engineering, enabling the creation of novel bioactive compounds that address critical challenges like antibiotic resistance and cancer. These innovations expand chemical space and optimize biosynthetic pathways, offering precise and scalable therapeutic solutions. Collaboration across synthetic biology, AI, and clinical research is essential to translating these breakthroughs into next‐generation treatments and revolutionizing drug discovery and patient care.

## AUTHOR CONTRIBUTIONS

The concept of the commentary was conceived by K.A.J.B. and E.F.B. Funding for the work was provided by K.A.J.B. and H.B.B. The manuscript was written by E.F.B. and K.A.J.B., with critical review and revisions provided by S.S. and H.B.B. All authors have read and approved the final version of the manuscript.

## CONFLICT OF INTEREST STATEMENT

HBB, SS, and KAJB are co‐founder of Myria Biosciences AG, for which SS and KAJB are CEO and CSO, respectively.

## ETHICS STATEMENT

Not applicable.
